# Ambient Exposure to Agricultural Pesticides during Pregnancy and Risk of Cerebral Palsy: A Population-Based Study in California

**DOI:** 10.3390/toxics8030052

**Published:** 2020-07-31

**Authors:** Zeyan Liew, Ondine S. von Ehrenstein, Chenxiao Ling, Yuying Yuan, Qi Meng, Xin Cui, Andrew S. Park, Peter Uldall, Jørn Olsen, Myles Cockburn, Beate Ritz

**Affiliations:** 1 Department of Environmental Health Sciences, Yale School of Public Health, New Haven, CT 06510, USA; 2 Yale Center for Perinatal, Pediatric, and Environmental Epidemiology, Yale School of Public Health, New Haven, CT 06510, USA; 3 Department of Community Health Sciences, Fielding School of Public Health, University of California, Los Angeles (UCLA), Los Angeles, CA 90095, USA; ovehren@ucla.edu; 4 Department of Epidemiology, Fielding School of Public Health, UCLA, Los Angeles, CA 90095, USA; lingcx@ucla.edu (C.L.); yuying.yn@gmail.com (Y.Y.); mengqixn@ucla.edu (Q.M.); asjpark@gmail.com (A.S.P.); britz@ucla.edu (B.R.); 5 Perinatal Epidemiology and Health Outcomes Research Unit, Division of Neonatology, Department of Pediatrics, Stanford University School of Medicine and Lucile Packard Children’s Hospital, Palo Alto, CA 94305, USA; cynthiacui1010@ucla.edu; 6 California Perinatal Quality Care Collaborative, Palo Alto, CA 94305, USA; 7 Department of Paediatrics, Copenhagen University Hospital, Rigshospitalet, 2100 Copenhagen, Denmark; pter.uldall@regionh.dk; 8 Department of Clinical Epidemiology, Aarhus University Hospital, 8000 C Aarhus, Denmark; jo@ph.au.dk; 9 Department of Preventive Medicine, Keck School of Medicine, University of Southern California, Los Angeles, CA 90033, USA; mylesc@usc.edu; 10 Department of Epidemiology, Colorado School of Public Health, University of Colorado, Aurora, CO 80045, USA; 11 Colorado Comprehensive Cancer Center, University of Colorado, Aurora, CO 80045, USA; 12 Department of Neurology, School of Medicine, UCLA, Los Angeles, CA 90095, USA

**Keywords:** pesticide, endocrine-disrupting chemical (EDC), cerebral palsy, movement disorders, neurodevelopment

## Abstract

Cerebral palsy (CP) is the most common neuro-motor disability in young children. Disruptions of maternal hormone function during pregnancy have been linked to CP risk. We investigated whether prenatal exposure to pesticide compounds with endocrine-disrupting action affect CP risk. We conducted a case-control study of 3905 CP cases and 39,377 controls born between 1998 and 2010 in California to mothers who lived in proximity (within 2 km) to any agricultural pesticide application recorded in the California Pesticide Use Reporting (PUR) system. We focused on 23 pesticides considered endocrine disruptors that are frequently used, and we found that exposure to any of the 23 pesticides in the first trimester was associated with elevated CP risks in female offspring (OR = 1.19; 95% CI: 1.05–1.35) but not males (OR = 0.99; 95% CI: 0.89–1.09) compared to the unexposed offspring. Positive associations were estimated for 15 pesticides suspected to affect the estrogen and 7 pesticides suspected to affect the thyroid hormone system. Our study suggests that first trimester exposure to pesticides that are suspected endocrine disruptors are associated with CP risk in female offspring. Pesticide exposures in early pregnancy may have sex-specific influences on the neuro-motor development of the fetus by interfering with endocrine systems.

## 1. Introduction

Cerebral palsy (CP) refers to a group of neurological disorders that appear in infancy or early childhood and that are attributed to lesions in the developing brain that permanently affect movement and muscle coordination [1]. CP affects about two-to-three per 1000 births [2,3], with a higher prevalence among infants born preterm [4]. CP is a lifelong condition, with more than half of the children with CP needing assistive devices such as walkers or wheelchairs, and many suffer from other major developmental disabilities including communication and behavioral problems, epilepsy, mental retardation, and secondary musculoskeletal problems [1,5]. The major CP subtypes are classified based on the type of motor impairment and areas of the brain affected, such as stiff muscles (spasticity), uncontrollable movements (dyskinesia), and poor balance and coordination (ataxia). Spastic CP is the most common subtype, affecting about 80% all CP cases [6]. In addition, CP cases can also be classified based on the distribution of limb involvement [1,6]. A lack of oxygen at birth (asphyxia) or head trauma are present in less than 10% of all CP cases [3], and the etiology of most CP cases remains unexplained [7,8].

CP has a complex and multifactorial etiology [3,4,5,7,9]. Several prenatal and perinatal risk factors such as multiple gestation, preterm birth, low birth weight, maternal infections, and maternal–fetal inflammatory responses have been associated with CP. [10] Maternal cigarette smoking during pregnancy has been found to be associated with CP risk in three larger pregnancy cohorts [11,12,13], but no associations have been found for alcohol intake [13] and total caffeine consumption during pregnancy [14]. Environmental risk factors for CP remain relatively unexplored, with only a few community-based or case-review studies suggesting that fetal in-utero exposure to methylmercury [15,16], low level lead exposure [17], and carbon monoxide exposure [18] may affect CP risk. It is also known that exposure to endocrine disruptors during critical periods of brain development can damage the nervous system and cause neurodevelopmental disorders [8,19,20]. 

Hormonal factors during pregnancy have been suggested to play a key role in CP etiology [19,20] and might partly explain higher CP prevalence in males than female [21,22]. Our group previously estimated sex-specific associations and observed a dose-response in CP risk among boys who were prenatally exposed to several perfluoroalkyl substances (PFAS) in the Danish National Birth Cohort [23]. PFAS are industrial pollutants in widespread use for which endocrine-disrupting effects have been documented in both experimental and epidemiological studies [24,25,26]. Similarly, some pesticides also act as endocrine disruptors and have developmental neurotoxic effects [27]. Pesticides can be ingested, inhaled, or dermally absorbed through contact with treated surfaces and exposure sources include food, water, air, dust, and soil. Population-based studies of pesticides have found that nearly all pregnant woman in the U.S. have measurable levels of pesticides in their blood [28]. California ranks first among US states in agricultural production, and many rural Californians live in close proximity to agricultural fields and have been shown to have pesticides in blood and urine [29], as they possibly are exposed through pesticide drift or the volatilization and spreading of pesticides through the air after application to fields [29,30]. Numerous studies conducted in California’s agricultural region have indicated that maternal proximity to pesticide applications during gestation were associated with increased risk of preterm birth [31], autism spectrum disorders [8,32], and reduced IQ scores during childhood [19]. Moreover, prenatal exposures to organophosphate [33,34], organochlorine [35], and carbamate pesticides [36] have been found to be related to poorer motor development in young children, but whether pesticides are also implicated in other more severe neuromotor disorders, such as CP, has not been studied. 

We conducted a population-based case-control study of CP in an agriculturally intensive region of California, United States, and we evaluated the associations between prenatal ambient exposures to agricultural pesticide compounds that may act as endocrine disruptors and the risk of CP. Maternal ambient exposure to specific pesticide compounds during pregnancy was estimated based on our geographic information system tool that integrates pesticide use records in the California state mandated Pesticide Use Reporting (PUR) program and relies on addresses from birth records for the study population [37,38]. The disease status of the child was ascertained through diagnostic records maintained at the California Department of Developmental Services (DDS). This population-based linkage design provided a unique opportunity to estimate prenatal pesticide exposure and CP risk while avoiding self-participation bias or recall bias for exposures.

## 2. Materials and Methods

This research was approved by the University of California, Los Angeles (UCLA), Office of the Human Research Protection Program (IRB#15-001651, date of approval 21 October 2015), and the California Committee for the Protection of Human Subjects (protocol #12-10-0861, procedure for this research was approved on 2 December 2016), and this study was exempted from informed consent requirements.

### 2.1. Study Sample

The California DDS provides services and support to individuals with developmental disabilities (including cerebral palsy and autism, among other conditions) living in the state of California at a total of 21 regional centers (https://www.dds.ca.gov/rc/listings/). In the DDS, CP is defined as follows: (1) a non-progressive lesion or disorder in the brain occurring during intrauterine life or the perinatal period and characterized by paralysis, spasticity, or the abnormal control of movement or posture that is manifest before 2 or 3 years of age, and (2) other significant motor dysfunction appearing before the age of 18 years. The primary outcome of interest of the study was having received a CP diagnosis. We also evaluated the sub-classifications of cases according to disease subtypes (spastic, hypotonic, and other) or the distribution of limb involvement in CP as unilateral (hemiplegia and monoplegia) or bilateral (quadriplegia and diplegia).

We identified 7530 CP cases born between 1998 and 2010 in the state of California based on diagnostic records maintained at the California DDS and linked these to California birth certificate records with a linkage success rate of 86.3%. This was accomplished with a probabilistic linkage based on child’s first name, last name, date of birth, sex, and both mother’s and father’s (whenever available) first name, last name, and date of birth. We estimated the probability that two records were for the same person by assigning a total linkage score generated for matches with the National Program of Cancer Registries Link PlusTM Software. We manually checked cases with borderline scores; the main reason for non-linkage was missing information on birth or DDS records.

Controls in this study were selected from a random subset of children born in 1998–2010 selected from California birth certificates. We first used a 1:10 ratio for cases to controls and also matched controls to the 7530 CP cases by sex and the exact birth year. These controls represented a subset of a larger control sample originally drawn for a study of autism [8], so none of the controls were allowed to have a diagnosis of CP or autism reported in DDS records by 2013.

CP risk is higher among multiple births [39], so we restricted the analyses to singletons only, leaving us with 6851 cases and 73,018 controls. Among these, 6341 case and 64,370 control birth addresses were successfully geocoded and had complete exposure information. These exclusions also included those birth addresses with a very low geocoding quality (i.e., at the USPS ZIP code area centroid level or coarser) in order to limit exposure misclassification. To assess agricultural pesticide exposures while reducing influences from unmeasured confounding due to other risk factors related to the geography of the residence [31], we further restricted the study sample to 3905 cases and 39,377 controls with a maternal residential address at birth in close proximity to agricultural pesticide applications; close proximity was defined as having any agricultural pesticide application recorded in the California’s PUR system within a 2 km buffer surrounding the maternal residence in the year of birth.

### 2.2. Pesticide Exposure Assessment 

We geocoded maternal residential addresses listed on the birth certificate using an automated approach [40], and we estimated residential ambient pesticide exposure using a geographic information system (GIS)-based Residential Ambient Pesticide Estimation System [37,38]. The model integrates California’s PUR, land use maps, and geocoded birth addresses to generate estimates of pesticide exposure during each month of pregnancy [31,38]. In brief, since 1974, agricultural pesticide applications for commercial use have been recorded in the PUR system, as mandated by the California Department of Pesticide Regulation (CDPR). Each PUR record includes the name of the pesticide’s active ingredient, the poundage applied, the crop type, and the location and date of application. The California Department of Water Resources (CDWR) performs countywide, large-scale surveys of land use and crop cover every 7–10 years. Land use maps increase spatial resolution because they provide more detailed land use geography that allowed us to more accurately determine where pesticides were applied [37]. Additional details on the exposure modeling have been presented previously [8,31,37,38].

Monthly exposures (pounds per acre) for each pesticide were calculated by summing the poundage of pesticide applied within a 2 km buffer surrounding each address and then weighting the total poundage by the proportion of acreage treated within the buffer. Previous pesticide studies have used different buffer sizes from 500 m to up to distances of 8000 m [41,42,43], depending on the pesticide of interest, landscape, and weather conditions. The choice of a 2 km (or 2000 m) buffer size was determined a priori and guided by our previous studies of pregnancy-related pesticide exposures and childhood disorders in the same study population [8,31]. We estimated the exposure for the first, second, and third trimesters defined as 0–12, 13–25, and 26–37 weeks of gestation, respectively. Since a higher proportion of CP cases are born preterm, the length of gestation and hence the exposure period was shorter among most cases than controls. To account for this, if a case was born preterm (<37 weeks or <259 days), each control was only assigned exposures for the same length of gestation as their matched case. For all term cases and matched controls, we assessed third trimester exposures during weeks 26–37. Cases and controls born before 26 weeks of gestation were not included in analyses that considered third trimesters exposures. 

We first limited our focus to frequently used chemicals defined as at least 5% exposure prevalence during pregnancy in the state of California in controls. We then selected pesticides with suspected endocrine-disrupting properties using the Pesticide Action Network (PAN) database and also relied on a systematic literature review conducted by McKinlay et al. [44]. For the PAN, we only considered pesticides listed as a potential endocrine-disrupting chemical (EDC) derived from governmental documents including the Illinois Environmental Protection Agency (EPA) list, the Danish EPA list, and the European Union (EU) prioritization list (for details, see http://www.pesticideinfo.org/Docs/ref_toxicity5.html (accessed Oct 2018)). A total of 32 pesticides identified from the PAN database were the frequently used chemicals in our population sample. Among these 32 pesticides, only 23 were also identified as potential EDCs in a literature review [44] that also referred to possible hormonal pathways (estrogen, androgen, or thyroid hormone) that each pesticide might affect (see Table S1).

### 2.3. Covariate 

Potential confounders were selected a priori according to the literature considering factors that might influence CP risk and also maternal exposure to pesticides during pregnancy [8,31,45]. We obtained information of maternal age at delivery (≤19, 20–24, 25–29, 30–34, and ≥35), maternal race/ethnicity (non-Hispanic White, Hispanic, Black/African American, Asian/Pacific Islander, and others), maternal education (<12 years, 12 years, 13–15 years, and ≥16 years), maternal parity (1, 2, and ≥3), maternal birthplace (US vs. non-US), and payment source for prenatal care from the birth certificate (private/health maintenance organization(HMO)/Blue Cross Blue Shield vs. MediCal/government/self-pay). The season of conception (fall, winter, spring, and summer defined as the months of October to December, January to March, April to June, and July to September, respectively) was calculated based on the last maternal menstrual period reported on the birth certificate. Neighborhood-level socioeconomic status (SES—a 5-level categorical variable from the lowest to the highest) was estimated based on census block group data [46].

### 2.4. Statistical Analysis

We conducted unconditional logistic regression analyses while adjusting for matching factors (child’s sex and birth year) to estimate the odds ratios (ORs) and 95% confidence intervals (CIs) for CP according to exposures. We first compared exposure to any of the 23 selected pesticide compounds in pregnancy with those unexposed to the 23 pesticides as the reference. Next, we evaluated the distribution of cumulative exposure by summing the numbers of different types of pesticides that the subjects were exposed to during pregnancy. We created three pre-defined exposure groups to assess the influence of the total number (count) of pesticides exposed as (1) unexposed to any of the 23 pesticides in pregnancy as a reference, (2) low-to-moderate exposure (from 1 to median counts), and (3) high exposure (above median counts). Analyses were repeated for exposure in each trimester first treating each exposure period separately, and then we performed an analysis that co-adjusted for all trimester exposures in the same model. Pesticides were further classified and analyzed according to three potential hormonal targets, i.e., the estrogen, androgen or thyroid hormone systems, as specified in the systematic literature review by McKinlay et al [44]. The analyses compared those ever exposed to any of the pesticides suspected to affect a specific hormonal system with those unexposed to these pesticides as the reference. Moreover, we estimated exposure effects for each pesticide compound individually, and we also performed sensitivity analyses with co-pollutant adjustments, including all 23 pesticides in the same model.

We adjusted for the matching variables (child’s sex and birth year) and maternal age at delivery, maternal race/ethnicity, maternal education, maternal birthplace, and the DDS regional center catchment area in the main model. We evaluated potential influences from maternal parity, season of conception, payment source for prenatal care as a proxy for family income, and neighborhood-level socioeconomic status in additional sensitivity analysis.

We stratified all analyses by sex to evaluate potential effect measure modifications [47]. Tests of heterogeneity (multiplicative scale) were performed by assessing the *p*-value of the interaction term for the exposure and child’s sex in the regression models. We performed analyses for CP sub-phenotypes according to those ever or never exposed to the 23 selected pesticide compounds in pregnancy and by trimesters. Gestational age at birth is strongly correlated with CP risk, and previous studies often stratified by preterm birth status to evaluate whether preterm infants were disproportionally affected [48]. However, gestational age at birth might be an intermediate variable between prenatal exposures and CP, and stratifying by preterm might induce a strong collider stratification bias [48,49]. Thus, instead of stratifying on preterm delivery, we classified CP as term (born ≥37 weeks) or preterm (born <37 weeks) cases and evaluated term or preterm CP as CP sub-phenotypes. All statistical analyses were performed using SAS 9.4 (SAS Institute Inc., Cary, NC, USA).

## 3. Results

Among the 3905 cases and 39,377 controls included in our study, about 57% of cases and controls were male (the sex ratio was 1.35 to 1), and about 60% were born in 1998–2004 (Table 1). Slightly more than half of the mothers included in this study were of Hispanic origin, about 44% were foreign born, and about 30% did not finish high school. The proportion of CP was higher among infants born preterm, and CP was also slightly more common with Black mothers and less common with mothers who completed college. The distribution of maternal birthplace being the US or foreign was similar in cases and controls. Among the 3905 CP cases, 2224 (60.0%) were classified as spastic, 613 (15.7%) as hypotonic, 1028 (26.3%) as other subtypes, and 40 (1.0%) as missing. Using a classification according to limb involvement, 604 (15.5%) cases were unilateral, 2781 (71.2%) were bilateral, and for 520 (13.3%), this information was missing.

### 3.1. Exposure to Suspected Endocrine-Disrupting Pesticides

In the total sample, we found that maternal first trimester exposures to any of the 23 pesticides with suspected endocrine-disrupting mechanism had a small increase in the odds (OR = 1.07) of CP in the offspring, but the 95% CI (0.99–1.16) crossed the null (Table 2). There were no associations with exposures in the second or the third trimester. In sex-stratified analyses, we estimated a 19% increase in the odds of CP in girls when mothers were exposed to any of the 23 pesticides during the first pregnancy trimester (OR = 1.19; 95% CI: 1.05–1.35), but no associations were found in boys in any trimester. The *p*-values for exposure and the sex interaction term were <0.01, 0.05, and 0.01 for the first, second, and third trimester exposure, respectively. The first trimester exposure effect estimate in girls was slightly strengthened (OR = 1.25; 95% CI: 1.07–1.46) in models that co-adjusted for exposures in other trimesters (Table 2). These results remained unchanged when additionally adjusting for other potential confounders such as neighborhood-level SES (Table S2).

Regarding the 23 pesticides, subjects were exposed to a median of five pesticides during pregnancy and three for each pregnancy trimester. The distribution of the cumulative count of pesticide exposure was similar for male and female offspring (Table S3). We did not find a linear dose–response pattern in analyses that assessed the number of pesticides; the strongest effects were estimated for the low-to-medium exposure group among girls exposed in the first trimester (Table S4).

### 3.2. Pesticides Classified According to Their Suspected Hormonal Targets

Pregnancy exposure to any of the seven pesticide compounds suspected to affect the thyroid hormone system was associated with CP risk in female offspring (OR = 1.14; 95% CI: 1.02–1.29), and risk estimates were elevated for first and third trimester exposures (Table 3). Moreover, first trimester exposure to any of the 15 pesticides with suspected estrogenic effects was also associated with elevated CP risk in female offspring (OR = 1.11; 95% CI: 1.00–1.25) (Table 3). No associations were found for suspected androgen interfering pesticides in female offspring, and the results for males were close to null for pesticides by hormonal system. 

### 3.3. Exposure to Individual Pesticide Compounds

Maternal first trimester exposure to dimethoate (OR = 1.17; 95% CI: 1.00–1.37), methomyl (OR = 1.19; 95% CI: 1.01–1.42), and diuron (OR = 1.24; 95% CI: 1.02–1.51) were positively associated with CP risk in female offspring (Table 4). Moreover, the estimated ORs for maternal first trimester exposure to malathion, mancozeb, fenarimol, and benomyl in female offspring were also elevated (in the range of 10–19%), but confidence intervals included the null. The positive effect estimates for exposure to these seven pesticide compounds in female offspring remained robust in mutually co-pollutant adjusted analyses with all 23 pesticides included in the same model. Some inverse associations were noted for exposure to specific pesticide compounds and risks in CP in male offspring, but these estimates became null in the co-pollutant adjusted models. 

### 3.4. CP Sub-Phenotypes

The estimated effect for maternal first trimester exposures to any of the 23 pesticides and risk of CP in female offspring were stronger for spastic CP (OR = 1.21; 95% CI: 1.02–1.43), the most common subtype that accounted for about 60% of all CP cases (Table S5). Using the classification according to ‘limbs affected,’ the estimated OR for first trimester exposure was slightly higher among bilateral (OR = 1.24; 95% CI: 1.07–1.44) than unilateral (OR = 1.11; 95% CI; 0.82–1.49) CP in girls (Table S6). Term CP cases in females had elevated risk estimates if exposed in the first trimester (OR = 1.24; 95% CI: 1.07–1.44). Among preterm CP cases, the point estimate in male offspring (OR = 1.16; 95% CI: 0.96–1.39) was increased, but the 95% confidence interval included the null (Table S7). 

## 4. Discussion

In this large population-based case-control study of CP among residents living in proximity of agricultural pesticide applications in California, maternal first trimester exposure to the most frequently applied pesticides that are suspected to act as endocrine disruptors was associated with increased CP risk in female offspring. In analyses of exposures categorized by suspected hormone target, positive associations were found for pesticides suspected to interfere with estrogen and thyroid hormone systems. Dimethoate, methomyl, and diuron were the individual pesticide compounds that were most consistently associated with CP risks in female offspring, and several additional pesticides (malathion, mancozeb, fenarimol, and benomyl) need further scrutiny. Given the large number of individual chemicals we evaluated, our results might be subject to chance error, and replication studies are needed. 

Maternal steroid and thyroid hormone anomalies during pregnancy [21,50,51] and gestational exposure to endocrine-disrupting compounds such as PFAS have been linked to CP risk in recent studies [23]. PFAS are persistent pollutants that can transfer from the mother to the fetus by passing through the placenta. [52,53] PFAS have neuroendocrine targets, including steroid and thyroid hormones [20,54], and they involve the maternal–fetal immune-response system that also has been associated with CP risk [55,56]. Many pesticides are suspected to be active endocrine disruptors in humans, and these compounds may interfere with hormone signaling pathways, either via direct binding with hormone receptors or inhibiting enzyme activities responsible for the biosynthesis of precursors of these hormones [27,57,58]. The thyroid hormone is necessary for the myelination of the nerve cells connecting the neurons of the brain stem, the basal ganglia, and the cerebellum—a process that is important for normal motor function development in humans [59]. Estrogens might protect the neonatal brain against hypoxic-ischemic injury, and sex-differences in susceptibility to injury have been suggested [21,60]. The influence that pesticides may have on estrogen-related susceptibility might be one explanation for the sex-specific associations we observed in our study. 

A non-causal explanation should also be considered for the sex-specific finding. CP is strongly associated with preterm and premature birth; thus, selection bias may arise, especially if an exposed fetus/infant with CP does not survive to be diagnosed [61]. This “live birth bias” might disproportionally impact results for male offspring. It is known that male fetuses are more susceptible to environment-induced fetal loss [62]. CP is more common among male infants [45], and male CP cases are also more likely to exhibit greater disease severity [22]. Therefore, if pesticide exposure results in a higher death rate among male fetuses/infants with CP, these cases would not be observed in our analyses and positive effect estimates might be biased towards the null or even beyond [61,63]. A previous study suggested that occupational and home pesticide exposures during the first trimester was associated with stillbirths and congenital anomalies in 10 California counties [64]. Whether ambient exposure to agricultural pesticides in California contribute to these outcomes is unknown and needs to be studied. 

A stronger association for CP was found in the medium exposure group when we relied on the count of the total number (out of 23 pesticides) to generate exposure for the first trimester. This may be explained by the well-characterized non-monotonicity of effects of chemicals on the endocrine system [55]. However, the analyses that relied on the number (count) of pesticides to generate an exposure measure did not consider the intensity or relative toxicity of each compound. Thus, they may not reflect an accurate exposure level or linear increase in exposure. It is known that the fetus is most susceptible to teratogen exposure effects in early gestation [65]. A previous study that investigated maternal infections or antibiotic use and CP risk in the offspring also reported the strongest associations in the first trimester [66]. Our own studies in the Danish cohort indicated that endocrine-disrupting compounds might interfere with the dynamics of maternal thyroid hormones in early gestation [67], and maternal thyroid function abnormalities were associated with adverse motor function in young children [68]. The early gestational period might be a critical time window for environmental exposures affecting CP risk. Exposure to three specific pesticides—dimethoate, diuron, and methomyl—was most consistently associated with CP risk in females. Dimethoate is an insecticide reported to disrupt the action of thyroid hormones and to increase blood concentrations of insulin while decreasing luteinizing hormone levels [69,70]. Diuron is a herbicide that has been shown to inhibit the action of androgens in animal models [71]. Methomyl, an insecticide, might promote aromatase activity and affect estrogen production [72]. Moreover, positive associations for first trimester exposure to the insecticide malathion and three fungicides (mancozeb, fenarimol, and benomyl) in female offspring were also noted, suggesting that various pesticide types and classes might target multiple hormonal pathways. Future studies to elucidate the mechanisms of actions for each of these pesticide compounds and their mixtures effects are needed to better understand how they may affect the development of CP and possibly other related neuro-motor disorders.

The strengths of our study include the large number of cases and controls that were available from the California state-wide birth and DDS registry. Our pesticide exposure assessment tool was developed based on California’s mandatory PUR program, which is known as the most detailed and comprehensive registry of pesticide use worldwide. We were able to use agricultural application records for specific pesticides with high spatial and temporal resolution to study trimester-specific exposure effects. This approach ruled out concerns about recall bias. Additionally, women would be expected to not be able to accurately report the specific type of agricultural pesticides being applied around their homes. Moreover, biomarker measures of multiple pesticide compounds, especially those with short biological half-life, would not be feasible in a population-based study of the size needed to investigate the risk of a rare disease such as CP. Evaluating pesticide compounds one at a time might miss potential mixture effects, especially if the pesticide compounds being evaluated affect similar biological pathways [73,74,75]. We thus examined associations related to combined exposures for a group of pesticides (i.e., exposure to any one of the selected endocrine disruptors) based on the PAN database coupled with a comprehensive systematic review [44]; we also examined associations related to individual pesticide compounds in single and multi-pollutant models. We were also able to evaluate CP sub-phenotypes based on detailed information recorded in the DDS registry. Our results were robust upon statistical adjustment for a range of potential confounders. 

Our study also had several limitations. One limitation was that we only had birth addresses available for geospatial estimation of exposure, and about 9–30% of families could have moved during pregnancy [76]. However, our recent study in the same study population compared address on birth records to residential histories from a public-record database (LexisNexis) [77]. This study suggested a moderate-to-strong correlation (r > 0.8) for frequently used pesticide compounds when comparing exposure estimates relying on birth address or using the LexisNexis data to account for moving. We also lacked exposure information on pesticides from other sources such as diet, home and garden pesticide use, and occupational exposure. The selection of suspected endocrine disruptors in our study was likely incomplete. The toxicologic assessment of agricultural pesticides in terms of endocrine disruptive function is still incomplete, and the direction the pesticides may have on hormone levels is hard to predict and may even differ by hormonal system. Moreover, other toxicity mechanisms such as oxidative stress possibly induced by pesticides were also not considered. The information available from the PUR system limited our ability to perform intensity of exposure or dose–response analyses. The reported poundage per acre sprayed for each pesticide does not reflect pesticide toxicity since a lesser amount of a more toxic chemical is expected to generate similar effects as a less toxic substance. We chose a 2 km buffer size based on previous research [8,31,78]. The potential misclassification of exposure was likely different for different chemicals since it not only depends on the buffer size but on the volatility, transport, and degradation of the agent that is related to meteorology and other factors that likely differ for each pesticide we investigated. These issues may have contributed to additional exposure misclassification, but all these sources of exposure misclassifications were likely to be non-differential by case and control status. 

Moreover, uncontrolled confounding by unmeasured or unknown confounders always remains a concern. Some of these potential confounders might include maternal occupation, body-mass-index, and lifestyle or dietary factors. However, our sex- and timing-specific findings for females suggest that any uncontrolled confounders would have to disproportionally affect female fetuses and particularly exposures in early pregnancy. Outcome misclassification was also possible, especially for the CP sub-phenotypes. A previous study estimated that, compared with the electronic records for autism in the California Department of Education, Special Education databases, 75–80% of all California children with autism are included in the DDS records [79]. The proportion of CP cases in California registered in the DDS has not been investigated. However, the potential under-registration of cases in DDS record was unlikely to create a large bias because—given the low disease prevalence—very few controls would have been true cases. We did not have sufficient clinical data to eliminate CP cases with well-established causes. The impacts of environmental exposures on CP cases without known causes might be even larger. The risk for selection bias was low in this record-linkage study because it was not influenced by self-selection for participation. The non-linkage (<14%) of case birth records and DDS records was due to missing values for the linkage variables, which was not expected to be strongly correlated with ambient exposures. Finally, our analyses were restricted to residents living near agricultural pesticide applications based on a 2 km buffer size, and this might limit the generalizability of the findings. Additionally, other pesticides such as those applied in the home or garden were not taken into account in our analyses.

## 5. Conclusions

Maternal first trimester exposures to ambient pesticides with suspected endocrine-disrupting action were associated with a small-to-moderate increase in CP risk for female offspring. Given the widespread and high usage of these pesticides in the agricultural regions of California, these findings raise concerns for the possible effects of ambient pesticide exposures on fetal brain development and the risk for neuro-motor disorders in childhood. Our findings for CP are novel and require replication.

## Figures and Tables

**Table 1:** Characteristics of the cases and controls selected from the study area in California, 1998–2010. CP: cerebral palsy.

Characteristics	CP Cases (N = 3905)		Controls (N = 39,377)	
	N	%	N	%
**Child’s sex**				
Male	2244	57.5	22,453	57.0
Female	1661	42.5	16,924	43.0
**Year of birth**				
1998–2004	2394	61.3	23,464	59.6
2005–2010	1511	38.7	15,913	40.4
**Gestational age (weeks)**				
<32	602	15.4	430	1.1
32–37	552	14.1	3074	7.8
37–42	2525	64.7	33,229	84.4
42–45	226	5.8	2644	6.7
**Maternal age (years)**				
Less than 20	436	11.2	4123	10.5
20–24	828	21.2	9149	23.2
25–29	979	25.1	10,498	26.7
30–35	894	22.9	9406	23.9
Greater than 35	768	19.7	6200	15.8
Missing	0	0	1	0
**Maternal education**				
Less than 8th grade	475	12.2	4441	11.3
9th–12th grade	734	18.8	7133	18.1
High school graduate/ high school diploma	1109	28.4	10,788	27.4
Degree less than college	822	21.1	8050	20.4
College or more than college	694	17.8	8146	20.7
Missing	71	1.8	819	2.1
**Maternal race/ethnicity**				
White, non-Hispanic	1115	28.6	11,653	29.6
Hispanic of any race	2112	54.1	20,813	52.9
Black	244	6.3	1920	4.9
Asian or Pacific islander	325	8.3	3733	9.5
Other race/ethnicity	109	2.8	1258	3.2
**Maternal birthplace**				
Foreign Born	1703	43.6	17,596	44.7
U.S. Born	2184	55.9	21,542	54.7
Missing	18	0.5	239	0.6

**Table 2:** Odds Ratio (OR) and 95% confidence interval (CI) for cerebral palsy according to pregnancy exposures to the 23 pesticides compounds and suspected endocrine disruptors.

Timing of Exposure	Total Sample				Female Offspring				Male Offspring			
	Cases (N = 3905)	Controls (N = 39,377)	OR ^1^ and 95% CI	OR ^3^ and 95% CI	Cases (N = 1661)	Controls (N = 16,924)	OR ^2^ and 95% CI	OR ^3^ and 95% CI	Cases (N = 2244)	Controls (N = 22,453)	OR ^2^ and 95% CI	OR ^3^ and 95% CI
	Exposure to any of the 23 pesticides											
1st trimester												
No	981	10,248	Ref	Ref	375	4424	Ref	Ref	606	5824	Ref	Ref
Yes	2924	29,129	1.07 (0.99, 1.16)	1.10 (1.00, 1.22)	1286	12,500	1.19 (1.05, 1.35)	1.25 (1.07, 1.46)	1638	16,629	0.99 (0.89, 1.09)	1.01 (0.89, 1.15)
2nd trimester												
No	1020	10,247	Ref	Ref	408	4406	Ref	Ref	612	5841	Ref	Ref
Yes	2885	29,130	1.01 (0.93, 1.09)	0.99 (0.89, 1.11)	1253	12,518	1.06 (0.94, 1.20)	0.96 (0.81, 1.13)	1632	16,612	0.98 (0.88, 1.08)	1.03 (0.89, 1.19)
3rd trimester ^4^												
No	1149	11,092	Ref	Ref	449	4779	Ref	Ref	700	6313	Ref	Ref
Yes	2578	25,612	0.98 (0.91, 1.06)	0.95 (0.86, 1.04)	1141	11,094	1.05 (0.93, 1.19)	0.99 (0.86, 1.15)	1437	14,518	0.93 (0.84, 1.03)	0.91 (0.81, 1.03)

^1^ Adjusted for child’s sex, birth year, maternal age, maternal education, maternal race, maternal birthplace, and Department of Developmental Services (DDS) regional center; ^2^ adjusted for birth year, maternal age, maternal education, maternal race, maternal birthplace, and DDS regional center; ^3^ additionally co-adjusted for each of the trimester exposure estimates in the same model; ^4^ subjects with gestational age <26 weeks were excluded in the assessment of 3rd trimester exposure.

**Table 3:** OR and 95% CI for cerebral palsy according to pregnancy exposures to the 23 pesticides compounds classified according to potential hormone target affected.

Timing of Exposure	Female Offspring			Male Offspring		
	Cases (N = 1661)	Controls (N = 16,924)	OR ^1^ and 95% CI	Cases (N = 2244)	Controls (N = 22,453)	OR ^1^ and 95% CI
Exposure to the 15 pesticides suspected to affect estrogen function ^2^						
During pregnancy						
No	398	4096	Ref	556	5353	Ref
Yes	1263	12,828	1.06 (0.93, 1.20)	1688	17,100	1.04 (0.94,1.16)
1st trimester						
No	568	6097	Ref	825	7944	Ref
Yes	1093	10,827	1.11 (1.00, 1.25)	1419	14,509	1.01 (0.92,1.11)
2nd trimester						
No	614	6114	Ref	838	7979	Ref
Yes	1047	10,810	0.99 (0.88, 1.10)	1406	14,474	1.00 (0.91,1.10)
3rd trimester ^3^						
No	639	6423	Ref	924	8312	Ref
Yes	951	9450	1.03 (0.92, 1.15)	1213	12,519	0.93 (0.84,1.02)
**Exposure to the 11 pesticides suspected to affect androgen function ^2^**						
During pregnancy						
No	368	4015	Ref	579	5434	Ref
Yes	1293	12,909	1.08 (0.95, 1.23)	1665	17,019	0.96 (0.86, 1.07)
1st trimester						
No	549	5987	Ref	843	7959	Ref
Yes	1112	10,937	1.09 (0.98, 1.23)	1401	14,494	0.94 (0.86, 1.03)
2nd trimester						
No	579	5991	Ref	877	8060	Ref
Yes	1082	10,933	1.02 (0.91, 1.14)	1367	14,393	0.91 (0.83, 1.00)
3rd trimester ^3^						
No	622	6364	Ref	920	8370	Ref
Yes	968	9509	1.01 (0.91, 1.14)	1217	12,461	0.93 (0.84, 1.02)
**Exposure to the 7 pesticides suspected to affect thyroid function ^2^**						
During pregnancy						
No	478	5558	Ref	767	7387	Ref
Yes	1183	11,366	1.14 (1.02, 1.29)	1477	15,066	0.93 (0.84, 1.02)
1st trimester						
No	693	7817	Ref	1079	10,267	Ref
Yes	968	9107	1.13 (1.01, 1.26)	1165	12,186	0.89 (0.81, 0.97)
2nd trimester						
No	723	7754	Ref	1066	10,275	Ref
Yes	938	9170	1.02 (0.91, 1.13)	1178	12,178	0.91 (0.83, 1.00)
3rd trimester ^3^						
No	733	7983	Ref	1105	10,398	Ref
Yes	857	7890	1.10 (0.99, 1.23)	1032	10,433	0.92 (0.84, 1.01)

^1^ Adjusted for birth year, maternal age, maternal education, maternal race, maternal birthplace, and DDS regional center; ^2^ information from McKinlay et al. 2008. Endocrine disrupting pesticides: Implications for risk assessment. Environ Int 34:168–183; ^3^ subjects with gestational age <26 weeks were excluded in the assessment of 3rd trimester exposure.

**Table 4:** OR and 95% CI for cerebral palsy according to first trimester exposure to each of the 23 pesticides compounds with suspected endocrine-disrupting properties.

Exposed in the First Trimester	Hormonal Target (I: Estrogen; II: Androgen; III: Thyroid) ^1^	Female Offspring				Male Offspring			
		Cases (N = 1661)	Controls (N = 16,924)	OR and 95% CI ^2^	OR and 95% CI ^3^	Cases (N = 2244)	Controls (N = 22,453)	OR and 95% CI ^2^	OR and 95% CI ^3^
Chlorpyrifos	II	581	5910	0.96 (0.86, 1.08)	0.93 (0.82, 1.06)	714	7859	0.91 (0.82, 1.00)	0.96 (0.86, 1.08)
Acephate	I, II, III	509	4965	0.97 (0.86, 1.09)	0.96 (0.83, 1.12)	656	6766	0.92 (0.83, 1.02)	0.99 (0.87, 1.12)
Malathion	III	443	4013	1.10 (0.97, 1.24)	1.13 (0.99, 1.30)	529	5361	0.95 (0.86, 1.06)	1.04 (0.92, 1.17)
Myclobutanil	I, II	364	3723	1.05 (0.93, 1.20)	1.06 (0.91, 1.24)	438	4960	0.92 (0.82, 1.03)	1.02 (0.89, 1.17)
Iprodione	I	375	3989	0.95 (0.84, 1.08)	0.90 (0.77, 1.06)	459	5384	0.87 (0.78, 0.98)	0.96 (0.83, 1.10)
Diazinon	I	356	3775	0.96 (0.85, 1.10)	0.92 (0.80, 1.07)	458	5192	0.88 (0.79, 0.99)	0.94 (0.83, 1.07)
Bifenthrin	II, III	334	3130	0.95 (0.83, 1.09)	0.94 (0.81, 1.10)	386	4207	0.86 (0.76, 0.97)	0.90 (0.79, 1.03)
Permethrin	I	307	3114	1.01 (0.87, 1.16)	1.00 (0.85, 1.19)	357	4153	0.91 (0.80, 1.04)	1.04 (0.90, 1.21)
Mancozeb	III	271	2508	1.10 (0.95, 1.27)	1.15 (0.97, 1.37)	320	3526	0.93 (0.82, 1.06)	1.05 (0.90, 1.22)
Dimethoate	I, III	238	2196	1.17 (1.00, 1.37)	1.18 (0.99, 1.41)	273	3116	0.95 (0.82, 1.09)	1.04 (0.89, 1.22)
Propiconazole	I, II	246	2377	0.99 (0.85, 1.16)	0.99 (0.83, 1.19)	263	3133	0.84 (0.72, 0.97)	0.92 (0.78, 1.08)
Simazine	I	169	1835	1.09 (0.90, 1.31)	1.02 (0.83, 1.26)	209	2407	1.03 (0.87, 1.21)	1.07 (0.89, 1.28)
Methomyl	I, II	195	1798	1.19 (1.01, 1.42)	1.19 (0.98, 1.45)	210	2523	0.91 (0.78, 1.07)	1.02 (0.85, 1.21)
Carbaryl	I	160	1649	0.98 (0.82, 1.17)	0.94 (0.78, 1.13)	205	2235	0.96 (0.82, 1.13)	1.04 (0.88, 1.23)
2,4-D, Dimethylamine Salt	II	157	1715	1.08 (0.90, 1.31)	1.07 (0.88, 1.30)	171	2170	0.84 (0.70, 1.00)	0.87 (0.72, 1.04)
Trifluralin	I	154	1727	0.96 (0.80, 1.15)	0.92 (0.76, 1.12)	194	2264	0.93 (0.79, 1.10)	1.00 (0.84, 1.19)
Lambda-Cyhalothrin	III	150	1705	0.96 (0.79, 1.16)	0.91 (0.73, 1.13)	174	2235	0.85 (0.71, 1.00)	0.94 (0.77, 1.14)
Diuron	II	153	1542	1.24 (1.02, 1.51)	1.26 (1.01, 1.57)	189	2102	1.06 (0.89, 1.26)	1.08 (0.89, 1.32)
Fenarimol	I, II	139	1435	1.12 (0.93, 1.36)	1.14 (0.93, 1.40)	152	1866	0.89 (0.74, 1.06)	0.94 (0.78, 1.14)
Triadimefon	I, II	126	1378	0.90 (0.73, 1.09)	0.87 (0.70, 1.08)	167	1820	0.88 (0.74, 1.05)	0.94 (0.78, 1.14)
Maneb	III	136	1467	1.05 (0.84, 1.30)	0.96 (0.75, 1.25)	142	2028	0.73 (0.60, 0.90)	0.80 (0.63, 1.01)
Dicofol	I, II	83	978	0.89 (0.70, 1.13)	0.83 (0.65, 1.07)	141	1423	1.01 (0.84, 1.21)	1.07 (0.88, 1.30)
Benomyl	I	123	1075	1.15 (0.93, 1.42)	1.11 (0.88, 1.39)	128	1552	0.83 (0.68, 1.01)	0.89 (0.72, 1.10)

^1^ Information from McKinlay et al. 2008. Endocrine disrupting pesticides: Implications for risk assessment. Environ Int 34:168–183; ^2^ adjusted for birth year, maternal age, maternal education, maternal race, maternal birthplace, and DDS regional center; ^3^ additionally co-adjusted for first trimester exposure to each of the 23 pesticides compounds in the same model.

## Supplementary Materials

The following are available online at https://www.mdpi.com/2305-6304/8/3/52/s1, Table S1: The list of pesticides included in the study, Table S2: Odds Ratio (OR) and 95% confidence intervals (CI) for CP according to pregnancy exposures to the 23 pesticides compounds and suspected endocrine disruptors, adjusting for additional potential confounders, Table S3: Distribution of the cumulative count of the 23 pesticides among the cases and the controls exposed, Table S4: Odds Ratio (OR) and 95% confidence interval (CI) for CP according to pregnancy exposures to the number (count) of the 23 pesticides compounds and suspected endocrine disruptors, Table S5: Odds Ratio (OR) and 95% confidence interval (CI) for CP subtypes according to pregnancy exposures to the 23 selected pesticides compounds and suspected endocrine disruptors, Table S6: Odds Ratio (OR) and 95% confidence interval (CI) for unilateral or bilateral CP cases according to prenatal exposures to pesticides and suspected endocrine disruptors, Table S7: Odds Ratio (OR) and 95% confidence interval (CI) for preterm and term CP cases according to prenatal exposures to pesticides and suspected endocrine disruptors.
