# Supplementary material for: Ambient Exposure to Agricultural Pesticides during Pregnancy and Risk of Cerebral Palsy: A Population-Based Study in California
Source: Toxics. 2020 Jul 31;8(3):52. doi: 10.3390/toxics8030052 (PMC7560316; doi:10.3390/toxics8030052)
Supplement: Supplementary file 1 [file toxics-08-00052-s001.pdf]

Supplementary Materials: Ambient Exposure to Agricultural Pesticides during Pregnancy and Risk of Cerebral Palsy: A Population-Based Study in California

Zeyan Liew, Ondine S. von Ehrenstein, Chenxiao Ling, Yuying Yuan, Qi Meng, Xin Cui, Andrew S. Park, Peter Uldall, Jørn Olsen, Myles Cockburn and Beate Ritz

Table S1. The list of pesticides included in the study.

Chemical Code [1]	Common Name	Prevalence of Exposure during Pregnancy [2]	Potential Hormone Target Affected [3]	Chemical Class [1]	Use Type [1]
253	Chlorpyrifos	35.0%	Androgen	Organophosphorus	Insecticide
1685	Acephate	29.8%	Estrogen, Androgen, Thyroid	Organophosphorus	Systemic insecticide
367	Malathion	23.8%	Thyroid	Organophosphorus	Insecticide, acaricide
2245	Myclobutanil	22.1%	Estrogen, Androgen	Azole	Fungicide
2081	Iprodione	23.8%	Estrogen	Dicarboximide	Fungicide
198	Diazinon	22.8%	Estrogen	Organophosphorus	Insecticide, acaricide
2300	Bifenthrin	18.6%	Androgen, Tyroid	Pyrethroid	Insecticide, acaricide
2008	Permethrin	18.5%	Estrogen	Pyrethroid	Insecticide
211	Mancozeb	15.3%	Thyroid	Dithiocarbamate, Inorganic-Z	Fungicide
216	Dimethoate	13.5%	Estrogen, Tyroid	Organophosphorus	Insecticide, acaricide
2276	Propiconazole	14.0%	Estrogen, Androgen	Azole	Fungicide
531	Simazine	10.8%	Estrogen	Triazine	Herbicide
383	Methomyl	11.0%	Estrogen, Androgen	N-Methyl Carbamate	Insecticide, acaricide
105	Carbaryl	9.9%	Estrogen	N-Methyl Carbamate	Insecticide
806	2,4-D, Dimethylamine Salt	9.9%	Androgen	Chlorophenoxy acid or ester	Herbicide
597	Trifluralin	10.1%	Estrogen	2,6-Dinitroaniline	Herbicide
2297	Lambda-Cyhalothrin	10.0%	Thyroid	Pyrethroid	Insecticide, acaricide
231	Diuron	9.3%	Androgen	Urea	Herbicide
1980	Fenarimol	8.4%	Estrogen, Androgen	Pyrimidine	Fungicide
2133	Triadimefon	8.1%	Estrogen, Androgen	Azole	Fungicide
369	Maneb	8.9%	Thyroid	Dithiocarbamate	Fungicide
346	Dicofol	6.1%	Estrogen, Androgen	Organochlorine	Acaricide
1552	Benomyl	6.7%	Estrogen	Benzimidazole	Fungicide

[1] Information from the PAN pesticide network http://www.Pesticideinfo.Org/list_chemicals.Jsp. [2] Estimated from 39,377 births selected at random from the birth certificates records in the State of California, 1998-2010. [3] Information synthesized from McKinlay et al. 2008. Endocrine disrupting pesticides: Implications for risk assessment. Environ Int 34:168-183.

Table S2. Odds Ratio (OR) and 95% confidence intervals (CI) for CP according to pregnancy exposures to the 23 pesticides compounds and suspected endocrine disruptors, adjusting for additional potential confounders.

Timing of Exposure	Total Sample		Female Offspring		Male Offspring	
	OR [1] and 95% CI	OR [2] and 95% CI	OR [3] and 95% CI	OR [4] and 95% CI	OR [3] and 95% CI	OR [4] and 95% CI
**Exposure to any of the 23 pesticides**						
During pregnancy						
No	Ref	Ref	Ref	Ref	Ref	Ref
Yes	1.06 (0.96, 1.17)	1.06 (0.95, 1.17)	1.09 (0.93, 1.28)	1.10 (0.94, 1.29)	1.04 (0.92, 1.19)	1.02 (0.90, 1.16)
1st trimester						
No	Ref	Ref	Ref	Ref	Ref	Ref
Yes	1.07 (0.99, 1.16)	1.05 (0.97, 1.14)	1.19 (1.05, 1.35)	1.19 (1.05, 1.36)	0.99 (0.89, 1.09)	0.96 (0.87, 1.07)
2nd trimester						
No	Ref	Ref	Ref	Ref	Ref	Ref
Yes	1.01 (0.93, 1.09)	1.01 (0.93, 1.09)	1.06 (0.94, 1.20)	1.07 (0.94, 1.22)	0.98 (0.88, 1.08)	0.97 (0.87, 1.07)
3rd trimester [5]						
No	Ref	Ref	Ref	Ref	Ref	Ref
Yes	0.98 (0.91, 1.06)	0.98 (0.91, 1.07)	1.05 (0.93, 1.19)	1.06 (0.94, 1.20)	0.93 (0.84, 1.03)	0.93 (0.84, 1.03)

[1] Adjusted for child's sex, birth year, maternal age, maternal education, maternal race, maternal birthplace and DDS regional center; [2] Adjusted for child's sex, birth year, maternal age, maternal education, maternal race, maternal birthplace, DDS regional center, parity, neighborhood-level socioeconomic status, season of conception, payment source for prenatal care, prenatal care in the first trimester; [3] Adjusted for birth year, maternal age, maternal education, maternal race, maternal birthplace and DDS regional center; [4] Adjusted for birth year, maternal age, maternal education, maternal race, maternal birthplace, DDS regional center, parity, neighborhood-level socioeconomic status, season of conception, payment source for prenatal care, prenatal care in the first trimester; [5] subjects with gestational age <26 weeks were excluded in the assessment of 3rd trimester exposure.

Table S3. Distribution of the cumulative count of the 23 pesticides among the cases and the controls exposed.

Timing of Exposure	Cumulative count of the 23 Pesticides among the Exposed								
	All Subjects			Cases			Controls		
	**Total**								
	**N**	**Median**	**25th–75th**	**N**	**Median**	**25th–75th**	**N**	**Median**	**25th–75th**
During pregnancy	36,834	5	2–9	3338	4	2–8	33,496	5	2–9
1st trimester	32,053	3	2–7	2924	3	2–6	29,129	4	2–7
2nd trimester	32,015	3	2–6	2885	3	1–6	29,130	3	2–7
3rd trimester	28,190	3	2–6	2578	3	1–6	25,612	3	2–6
	**Female offspring**								
	**N**	**Median**	**25th–75th**	**N**	**Median**	**25th–75th**	**N**	**Median**	**25th–75th**
During pregnancy	15,863	5	2–9	1438	4	2–8	14,425	5	2–9
1st trimester	13,786	3	2–6	1286	3	2–6	12,500	4	2–7
2nd trimester	13,771	3	2–6	1253	3	2–6	12,518	3	2–6
3rd trimester	12,235	3	2–6	1141	3	2–6	11,094	3	2-6
	**Male offspring**								
	**N**	**Median**	**25th–75th**	**N**	**Median**	**25th–75th**	**N**	**Median**	**25th–75th**
During pregnancy	20,971	5	2–9	1900	4	2–7	19,071	5	2–9
1st trimester	18,267	3	2–7	1638	3	2–6	16,629	4	2–7
2nd trimester	18,244	3	2–7	1632	3	1–6	16,612	3	2–7
3rd trimester	15,955	3	2–6	1437	3	1–6	14,518	3	2–6

Table S4. Odds Ratio (OR) and 95% confidence interval (CI) for CP according to pregnancy exposures to the number (count) of the 23 pesticides compounds and suspected endocrine disruptors.

Timing of Exposure	Total Sample			Female Offspring			Male Offspring		
	Cases (N = 3905)	Controls (N = 39,377)	OR [1] and 95% CI	Cases (N = 1661)	Controls (N = 16,924)	OR [2] and 95% CI	Cases (N = 2244)	Controls (N = 22,453)	OR [2] and 95% CI
**Numbers (count) of the 23 pesticides exposed [3]**									
During pregnancy									
0	567	5881	Ref	223	2499	Ref	344	3382	Ref
1 to 5	2020	18,854	1.10 (0.99, 1.22)	854	8185	1.11 (0.95, 1.31)	1166	10,669	1.10 (0.99, 1.22)
>5	1318	14,642	1.00 (0.89, 1.11)	584	6240	1.05 (0.88, 1.25)	734	8402	0.99 (0.89, 1.11)
1st trimester									
0	981	10,248	Ref	375	4424	Ref	606	5824	Ref
1 to 3	1600	14,532	1.14 (1.04, 1.24)	701	6246	1.27 (1.11, 1.46)	899	8286	1.14 (1.04, 1.24)
>3	1324	14,597	0.98 (0.90, 1.08)	585	6254	1.09 (0.95, 1.27)	739	8343	0.98 (0.90, 1.08)
2nd trimester									
0	1020	10,247	Ref	408	4406	Ref	612	5841	Ref
1 to 3	1564	14,707	1.05 (0.96, 1.15)	652	6356	1.06 (0.93, 1.22)	912	8351	1.05 (0.96, 1.15)
>3	1321	14,423	0.96 (0.87, 1.05)	601	6162	1.05 (0.91, 1.21)	720	8261	0.96 (0.87, 1.05)
3rd trimester [4]									
0	1149	11,092	Ref	449	4779	Ref	700	6313	Ref
1 to 3	1481	13,859	1.02 (0.93, 1.10)	643	6068	1.07 (0.94, 1.23)	838	7791	1.01 (0.93, 1.10)
>3	1097	11,753	0.93 (0.85, 1.02)	498	5026	1.03 (0.89, 1.19)	599	6727	0.93 (0.85, 1.02)

[1] Adjusted for child's sex, birth year, maternal age, maternal education, maternal race, maternal birthplace and DDS regional center; [2] Adjusted for birth year, maternal age, maternal education, maternal race, maternal birthplace and DDS regional center; [3] classified as none, =< median, > median based on the exposure distribution from all subjects; [4] Subjects with gestational age at birth < 26 weeks were excluded in the assessment of 3rd trimester exposure.

Table S5. Odds Ratio (OR) and 95% confidence interval (CI) for CP subtypes according to pregnancy exposures to the 23 selected pesticides compounds and suspected endocrine disruptors.

Timing of Exposure	Spastic CP				Hypotonia CP				Other types [2]			
	Female only		Male only		Female only		Male only		Female only		Male only	
	Cases (N = 932)	OR [1] and 95% CI	Cases (N = 1292)	OR [1] and 95% CI	Cases (N = 280)	OR [1] and 95% CI	Cases (N = 333)	OR [1] and 95 %CI	Cases (N = 430)	OR [1] and 95% CI	Cases (N = 598)	OR [1] and 95% CI
						Exposure to any of the 23 pesticides						
During pregnancy												
No	122	Ref	199	Ref	42	Ref	60	Ref	56	Ref	82	Ref
Yes	810	1.12 (0.91, 1.38)	1093	1.07 (0.91, 1.27)	238	0.96 (0.68, 1.37)	273	0.84 (0.63, 1.13)	374	1.14 (0.84, 1.54)	516	1.13 (0.88, 1.44)
1st trimester												
No	209	Ref	350	Ref	66	Ref	101	Ref	95	Ref	148	Ref
Yes	723	1.21 (1.02, 1.43)	942	1.01 (0.89, 1.16)	214	1.12 (0.84, 1.50)	232	0.83 (0.65, 1.06)	335	1.23 (0.96, 1.58)	450	1.05 (0.86, 1.28)
2nd trimester												
No	228	Ref	355	Ref	65	Ref	99	Ref	112	Ref	151	Ref
Yes	704	1.07 (0.91, 1.26)	937	0.99 (0.87, 1.13)	215	1.14 (0.85, 1.53)	234	0.87 (0.68, 1.12)	318	0.96 (0.76, 1.22)	447	1.04 (0.85, 1.26)
3rd trimester [3]												
No	243	Ref	400	Ref	83	Ref	113	Ref	120	Ref	181	Ref
Yes	641	1.10 (0.93, 1.29)	827	0.95 (0.84, 1.09)	188	0.95 (0.72, 1.25)	208	0.84 (0.66, 1.07)	297	1.02 (0.81, 1.28)	389	0.94 (0.78, 1.14)

[1] Adjusted for birth year, maternal age, maternal education, maternal race, maternal birthplace and DDS regional center; [2] Includes dyskinesia, ataxic, and unclassified; [3] subjects with gestational age <26 weeks were excluded in the assessment of 3rd trimester exposure.

Table S6. Odds Ratio (OR) and 95% confidence interval (CI) for unilateral or bilateral CP cases according to prenatal exposures to pesticides and suspected endocrine disruptors.

Timing of Exposure	Unilateral CP				Bilateral CP			
	Female Only		Male Only		Female Only		Male Only	
	Cases (N = 279)	OR [1] and 95%CI	Cases (N = 325)	OR [1] and 95%CI	Cases (N = 1175)	OR [1] and 95%CI	Cases (N = 1606)	OR [1] and 95%CI
**Exposure to any of the 23 pesticides**								
During pregnancy								
No	34	Ref	52	Ref	163	Ref	241	Ref
Yes	245	1.14 (0.78, 1.66)	273	0.96 (0.69, 1.32)	1012	1.06 (0.88, 1.27)	1365	1.07 (0.92, 1.24)
1st trimester								
No	64	Ref	89	Ref	259	Ref	428	Ref
Yes	215	1.11 (0.82, 1.49)	236	0.95 (0.73, 1.23)	916	1.24 (1.07, 1.44)	1178	1.00 (0.89, 1.13)
2nd trimester								
No	74	Ref	86	Ref	285	Ref	433	Ref
Yes	205	0.90 (0.68, 1.20)	239	0.98 (0.75, 1.27)	890	1.08 (0.93, 1.25)	1173	1.00 (0.88, 1.12)
3rd trimester [2]								
No	68	Ref	107	Ref	322	Ref	491	Ref
Yes	199	1.16 (0.87, 1.55)	202	0.81 (0.63, 1.04)	801	1.03 (0.90, 1.19)	1040	0.95 (0.85, 1.07)

[1] Adjusted for birth year, maternal age, maternal education, maternal race, maternal birthplace and DDS regional center; [2] subjects with gestational age <26 weeks were excluded in the assessment of 3rd trimester exposure.

Table S7. Odds Ratio (OR) and 95% confidence interval (CI) for preterm and term CP cases according to prenatal exposures to pesticides and suspected endocrine disruptors.

Timing of Exposure	Preterm CP				Term CP			
	Female only		Male only		Female only		Male only	
	Cases (N = 429)	OR [1] and 95%CI	Cases (N = 725)	OR [1] and 95%CI	Cases (N = 1232)	OR [1] and 95%CI	Cases (N = 1519)	OR [1] and 95%CI
**Exposure to any of the 23 pesticides**								
During pregnancy								
No	62	Ref	102	Ref	161	Ref	242	Ref
Yes	367	0.96 (0.72, 1.29)	623	1.11 (0.89, 1.38)	1071	1.14 (0.95, 1.37)	1277	1.01 (0.87, 1.18)
1st trimester								
No	101	Ref	171	Ref	274	Ref	435	Ref
Yes	328	1.08 (0.85, 1.37)	554	1.16 (0.96, 1.39)	958	1.24 (1.07, 1.44)	1084	0.92 (0.81, 1.04)
2nd trimester								
No	101	Ref	182	Ref	307	Ref	430	Ref
Yes	328	1.11 (0.88, 1.41)	543	1.05 (0.88, 1.26)	925	1.04 (0.90, 1.20)	1089	0.94 (0.83, 1.07)
3rd trimester [2]								
No	113	Ref	224	Ref	336	Ref	476	Ref
Yes	245	0.89 (0.70, 1.13)	394	0.76 (0.64, 0.91)	896	1.11 (0.97, 1.28)	1043	1.01 (0.90, 1.14)

[1] Adjusted for birth year, maternal age, maternal education, maternal race, maternal birthplace and DDS regional center; [2] subjects with gestational age <26 weeks were excluded in the assessment of 3rd trimester exposure.
